# Using radial pulse wave as hemodynamic measurements to quantify effects of acupuncture therapy for patients with traumatic brain injury and ischemia stroke

**DOI:** 10.1016/j.jtcme.2022.08.005

**Published:** 2022-09-01

**Authors:** Jhong-Kuei Chen, Wan-Ting Tsai, Shinn-Zong Lin, Sheng-Hung Wang, Gin-Chung Wang, Tien-Chung Wang, Hao-Ping Chen, Tsung-Jung Ho

**Affiliations:** a Department of Chinese Medicine, Hualien Tzu Chi Hospital, Hualien, Taiwan; b Institute of Medical Sciences, Tzu Chi University, Hualien, Taiwan; c Integration Center of Traditional Chinese and Modern Medicine, Hualien Tzu. Chi Hospital, Hualien, Taiwan; d Department of Neurosurgery, Hualien Tzu Chi Hospital, Hualien, Taiwan; e MiiAnn Medical Research Center, Taipei, Taiwan; f JinMu Health Technology, Taipei, Taiwan; g Seoul National University- Korea, Department of Food and Nutrition, Seoul, South Korea; h Department of Bio-chemistry, Tzu Chi University, Hualien, Taiwan

**Keywords:** Traumatic brain injury, Acupuncture, Stroke, Harmonic analysis, Pulse wave analysis

## Abstract

**Background and aim:**

Traumatic Brain Injury (TBI) and stroke are major sources of death and disability worldwide. Acupuncture has been used as a supplemental therapy for patients with TBI and stroke. This study was aimed to evaluate the effects of acupuncture therapy for patients with TBI and stroke by radial pulse spectrum.

**Experimental procedure:**

22 patients (6 TBI and 16 stroke) were enrolled and underwent radial pressure wave measurement before and after acupuncture treatment at Dubi (ST-35), Zusanli (ST-36) and Jiexi (ST-41). The harmonic analysis of the radial pressure wave was calculated and transformed into Fourier series coefficients Cn, Pn and the variation coefficient CnCV.

**Results:**

After acupuncture, systolic blood pressure, heart rate, and Glasgow Coma Scale changed very slightly. The harmonic index C4, C7, C9, C10, C3CV and C5CV had significant increases. (P < 0.05) After 3-week course acupuncture treatment, systolic blood pressure, C7, C8, C9, C10 and P10 had significant increases. (P < 0.05)

**Conclusion:**

Harmonic analysis of radial pulse waves may detect earlier circulatory system changes of acupuncture treatment before they were evident with other hemodynamic readings or scale.

## Highlights

• The pressure pulse spectrum of the radial pressure wave of patients with traumatic brain injury and ischemia stroke after acupuncture therapy may gradually change.
• Harmonic analysis of the pressure pulse is a useful method to quantitatively study and quantify the efficacy of acupuncture treatment on traumatic brain injury and stroke patient.
• Harmonic analysis may understand the underlying mechanisms of cardiovascular changes in patients with acupuncture treatment.

## Graphical abstract

**Figure:**
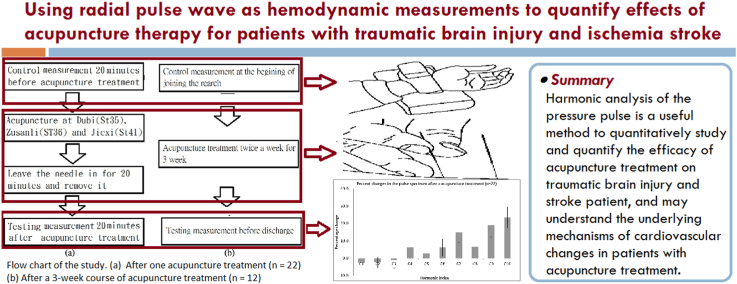

## List of abbreviations

TBI: Traumatic Brain Injury
Cn: The nth harmonic proportions
CnCV: The variation coefficient of nth harmonic amplitude
GCS: Glasgow Coma Scale
SBP: Systolic blood pressure
DBP: Diastolic blood pressure
HR: Heart rate

## 1 Introduction

Traumatic Brain Injury (TBI) and stroke are major sources of death and disability worldwide. Complementary to conventional treatments such as surgery, medication, and rehabilitation, acupuncture is increasing a supplemental therapy for patients with TBI, ischemia stroke.^1^^,^^2^ The Glasgow Coma Scale (GCS) is widely used in the assessment of clinical severity and prediction of outcome after TBI.^3^ The GCS can be divided into three parts, including eye open, verbal response and motor response, and is a relatively simple and quick way to assess the severity of a patient with traumatic brain injury in a clinical setting.^3^

The resonance theory provides a scientific explanation of the acupuncture effect^4^ and the meridians can be classified according to its effects on the pulse spectrum from the hemodynamic perspective.^5^ An established PR Wave model has illustrated the relationship between radial pulse waves and the cardiovascular system.^6^ There are already clinical applications in a number of areas such as acupuncture, Chinese herbal medicine, hypertension medication, liver injury, and end-of-life procedures.^5^

Acupuncture research has been the focus of long-standing and persistent attacks by skeptics.^7^ The pulse spectrum hemodynamic measurements ensured the specific frequency effect and acupuncture needle was applied on the acupuncture points.^5^ According to the clinical statistics of Hualien Tzu-Chi Hospital, the survival rate of TBI or stroke patients with acupuncture treatment for one year was twice as high as that without acupuncture treatment, and the survival rate for two years was increased to three times. Therefore, in this study, we specifically observed patients with TBI or stroke who had acupuncture treatment and investigated the quantitative radial pulse wave variations with the widely recognized outcome measurements such as GCS on the effects of post-surgery acupuncture therapy. The result of this study may also help pave the way to a more standardized acupuncture treatment protocol based on the hemodynamic measurements.

## 2 Materials and methods

### 2.1 Study population

Patients with TBI and stroke were recruited from the Hualien Tzu Chi General Hospital in Taiwan from April to August in 2020. The cohort included 13 men (59%) and 9 women (41%), aged between 28 and 85. The study was conducted in accordance with the Helsinki Declaration and Good Clinical Practice Guidelines approved by the Hualien Tzu Chi General Hospital Institutional Review Board (IRB number: IRB108-200-B). Patients were hospitalized immediately after the injury (n = 6) or the stroke (n = 16) and on average received 3 weeks of treatment in the hospital.

### 2.2 Study design

**Fig. 1:**
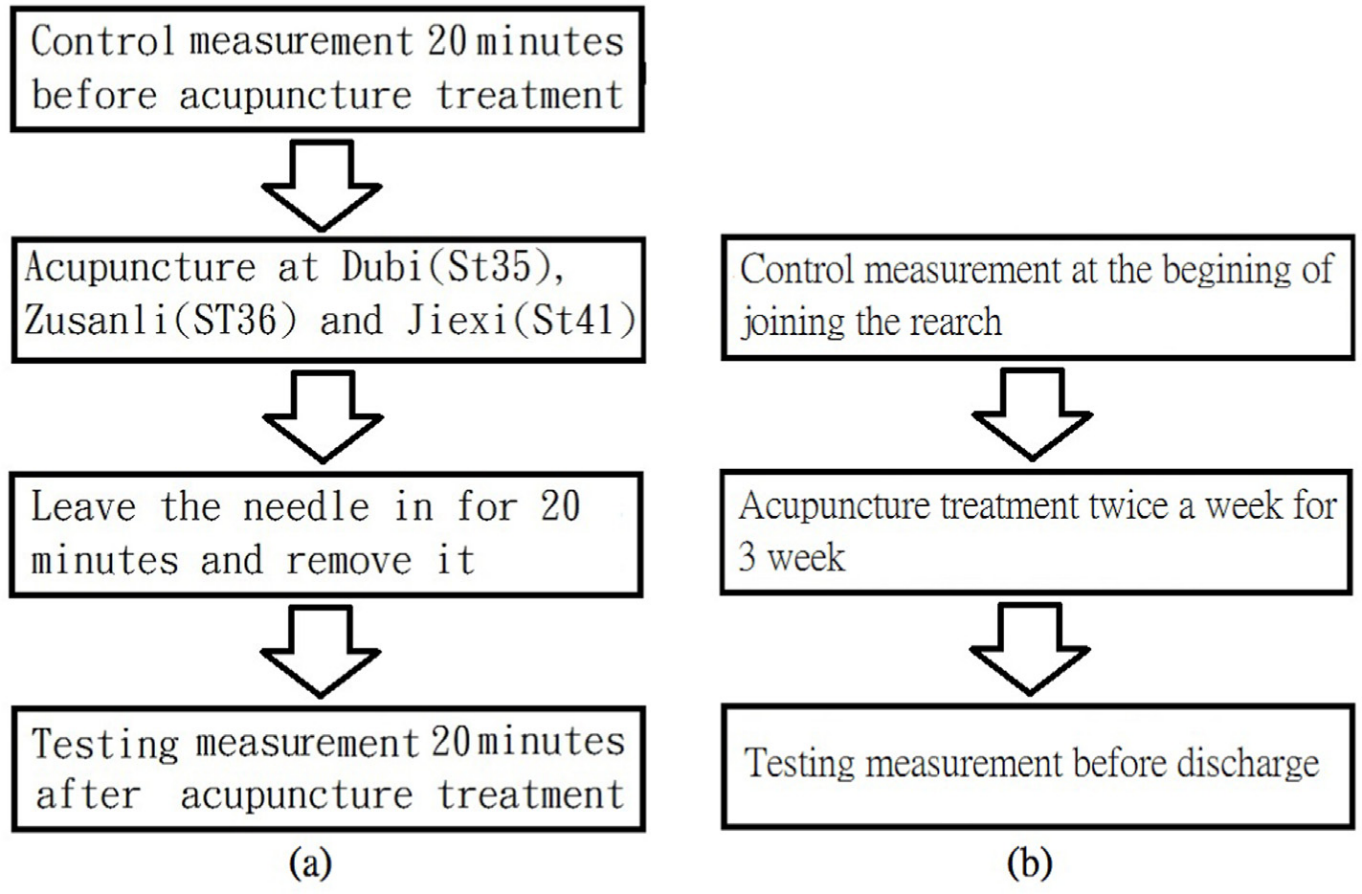
Flow chart of the study. (a) After one acupuncture treatment (n = 22) (b) After a 3-week course of acupuncture treatment (n = 12).

The acupuncture treatments were at Dubi (ST-35), Zusanli (ST-36) and Jiexi (ST-41) according to the clinical experience of the specialist. As demonstrated in Fig. 1, radial pulse spectrum was evaluated for the immediate 20 min after each treatment and after 3 weeks of treatments. The radial pulse spectrum of each of the 22 patients (6 TBI and 16 stroke) was measured prior to and 20 min post the removal of the needles. 12 patients (4 TBI and 8 stroke), 8 males and 4 females, completed a course of 3-week acupuncture treatments which including 3 times acupuncture treatments a week for 3 weeks. Their radial pulse spectrum was measured again to observe the long-term effect of the acupuncture treatments.

Radial pulse spectrum was non-invasively measured by a medical grade device (TD01C, MII-ANN Technology, Taiwan). After completing the radial pulse wave measurement, blood pressures and heart rate were measured by an automatic blood pressure monitor (CARESCAPE V100, GE Healthcare, USA) and GCS was evaluated by a physician.

### 2.3 Data analysis

The continuous pressure pulses were recorded for 12-s. The sampling rate of pressure data was 400 points per second. For example, with a heart rate of 100 beats per minute (1.67 Hz), the resolution of the sensor in the 10th phase is 0.262 radians. The pulse waveforms were transformed into the frequency domain. Heart beat is a repeated signal with period T, the pulse (P(t)) can be decomposed into harmonics as following:^8^

(1) $$P\left(t\right)=A0+\sum_{n=1}^{N}An\cos\left(\frac{2\pi n}{T}t+\varphi n\right)$$

In this study, we focused on the change in the first ten harmonics and computed the harmonic proportions (Cn, where n = 1–10) instead of the amplitude (An, where n = 1–10). Cn was defined as Cn = An/A0, where A0 is the mean pulse pressure value. Pn was the nth phase of the harmonic. The variation coefficient of nth harmonic amplitude (CnCV) are defined in the supplementary (as shown in Fig. S1).

The radial pulse wave measurement before each acupuncture treatment was used as control data. The effect of the acupuncture treatment on the Cn, is presented as the percentage change, %C^Cn^, in the experiment between the acupuncture treatment and control conditions as follows:

(2) $$\left(\%C^{Cn}\right)=100\times \frac{Cn,test-Cn,cont}{Cn,cont}$$

where Cn, cont is the Cn of the control, and Cn, test is the Cn of the test pulse waveform.

### 2.4 Statistical analysis

Indices such as systolic blood pressure (SBP), diastolic blood pressure (DBP), and heart rate (HR) are reported as mean ± standard deviation. Student's t-test was used for statistical comparisons. The level of statistical significance was set at P < 0.05.

## 3 Results and discussion

**Fig. 2:**
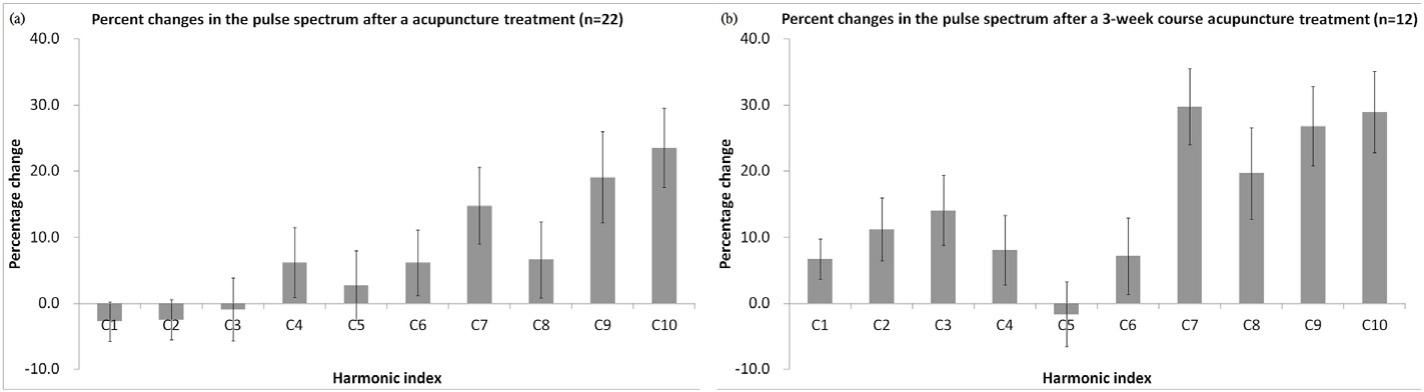
Percent changes in the pulse spectrum (a) after one acupuncture treatment and (b) after a 3-week course of acupuncture treatment.

Fig. 2 showed the change of %C^Cn^ after (a) one acupuncture treatment and (b) a 3-week course of acupuncture treatment. This study provided a non-invasive measurement to evaluate the changes in the physiological and circulatory system of patients with TBI and stroke. Harmonic analysis of radial pulse wave has been found to characterize physiological meanings beyond blood pressure.^9^ The study design adopted self-comparison method,^10^ and found significant changes in the harmonic indexes. The self-comparison method evaluates the changes of the same patient before and after a treatment avoiding individual variations that may be greater than the effect of the treatment.

Table 1 displayed the clinical characteristics and harmonic indexes of patients before and after acupuncture treatments. Previous studies have found that acupuncture altered the state of blood pressure harmonics.^4^ The similar effect was found in this trial. C4, C7, C9 and C10 were significantly increased after acupuncture. Other studies have pointed out the use of acupuncture improved the state of stroke patients.^11^ Acupuncture at Zusanli (ST-36) also had been shown to have a good healing effect on acute ischemic stroke.^12^ In this study, the patients self-reported improvement of the conditions, however, GCS was not significantly increased immediately after acupuncture at Dubi (ST-35), Zusanli (ST-36) and Jiexi (ST-41).

**Table 1:** The clinical characteristics and harmonic index of patients with an acupuncture treatment.

	Pre-acupuncture	Post- acupuncture
Demographic characteristics		
N	22	
Age (years)	58.0 ± 21.9	
Male (%)	13 (59%)	
BMI (kg/m2)	22.1 ± 3.5	
Blood Pressure index		
SBP (mm-Hg)	139 ± 25	137 ± 21
DBP (mm-Hg)	70 ± 11	71 ± 10
HR (bpm)	84 ± 18	82 ± 18
Glasgow Coma Scale	11.5 ± 3.5	11.7 ± 3.5
Harmonic index		
C1	0.958 ± 0.024	0.945 ± 0.029
C2	0.527 ± 0.022	0.532 ± 0.032
C3	0.258 ± 0.014	0.267 ± 0.015
C4	0.163 ± 0.009	0.176 ± 0.008 a
C5	0.148 ± 0.010	0.156 ± 0.010
C6	0.089 ± 0.010	0.099 ± 0.010
C7	0.060 ± 0.007	0.069 ± 0.007 a
C8	0.046 ± 0.005	0.050 ± 0.006
C9	0.032 ± 0.005	0.037 ± 0.004 a
C10	0.023 ± 0.004	0.029 ± 0.004 a
Variation coefficient of Harmonic index		
C1CV	4.660 ± 0.607	6.232 ± 1.187
C2CV	5.830 ± 0.722	6.748 ± 0.912
C3CV	9.649 ± 0.912	13.248 ± 1.838 a
C4CV	9.807 ± 1.453	11.973 ± 1.867
C5CV	8.320 ± 1.118	12.797 ± 1.847 a
C6CV	13.054 ± 1.917	16.402 ± 2.346
C7CV	15.656 ± 1.912	14.626 ± 1.532
C8CV	14.409 ± 1.558	16.497 ± 2.357
C9CV	18.224 ± 2.533	16.943 ± 2.389
C10CV	21.723 ± 3.024	17.805 ± 2.482

BMI= Body Mass Index, SBP= Systolic blood pressure, DBP = Diastolic blood pressure, HR = heart rate. Cn = nth Harmonic proportions, CnCV = variation coefficient of nth harmonic amplitude.

a P < 0.05 compared with control.

The evidence provided from clinical and laboratory suggests that acupuncture induces multi-level regulation via complex mechanisms to explain the beneficial effects against cerebral ischemia and TBI.^13^ Acupuncture not only activated relevant brain regions,^14^ modulated cerebral blood flow^15^ and related molecules,^16^ but also promotes neurogenesis, angiogenesis as well as neuroplasticity after ischemic damage.^17^ Evidence from laboratory indicated that acupuncture improve neurological recovery after TBI by activating Brain-derived neurotrophic factor and tropomyosin receptor kinase B pathway.^18^ Table S1 illustrated the clinical characteristics and harmonic indexes of the 12 patients who completed the 3-week acupuncture treatments. After 3 weeks of acupuncture treatments, GCS increased in 7 out of 12 patients, and C7, C8, C9 and C10 also significantly increased. The harmonic analysis in addition quantified the physiological changes of acupuncture from the blood circulation system point of view.

TBI and stroke are related diseases caused by cerebral ischemia from trauma or vascular obstruction. High-frequency harmonic indicators were found to be related to brain circulation. Changes in C6–C10 were observed after drinking tea to boost the brain circulation.^19^ Brain complications were related to C6CV in patients with type 2 diabetes.^20^ Changes and correlation were identified between brain waves and C6 and C9 during sleep.^21^ These high frequency harmonics are also related to the head meridian of Chinese medicine, including C5 stomach meridian, C6 gallbladder meridian, C7 bladder meridian, C8 large intestine meridian, C9 sanjiao meridian, and C10 small intestine meridian.^22^ In this study, C4, C7, C9, C10, C3CV and C5CV significant increased after acupuncture treatment, and acupuncture treatment may improve the brain circulation of patients.

CnCV represents the unstable state of the circulatory system, and changes in CnCV may also be used to evaluate the effect of treatment.^23^ Fig. S1 illustrates the mathematical definition of CnCV. The CnCV increases when the organ is severely damaged or is failing, because the circulatory system is no longer able to maintain the resonance.^24^, ^25^, ^26^ This study found that C3CV and C5CV increased after the acupuncture treatment and hypothetically these changes may be related to the increased blood circulation.

After 3 weeks of acupuncture treatment, P10 increased significantly. Mathematically, the phase of the pulse spectrum represents the starting position of each harmonic. In our previous research, we found that the harmonic phase changes significantly when the organ is seriously injured.^27^, ^28^, ^29^ We analyzed the phase of 12 patients diagnosed with TBI and stroke suffering severe head injury, and completed 3 weeks of acupuncture treatment. Among these patients, the 7 patients with increased GCS had a significant difference in P7 (2.225 ± 0.469 vs. 1.269 ± 0.229, p < 0.05). This result indicated that the harmonic may be potentially used as a tool to assess the organ damages.

However, it is regrettable that real-time head blood flow information was not obtained for reference in this study. In future work, through real brain blood flow measurements such as laser Doppler, ultrasound, fMRI and nuclear imaging, it will be possible to clarify the effect of acupuncture on brain circulation in TBI and stroke patients and the relationship between each harmonic index and head circulation. In addition, although there was no immediate significant difference in GCS in this study, the hemodynamic mechanisms of the three GCS assessments-eye opening, verbal response, and motor response-could be investigated separately through harmonic analysis after accumulating enough patient data.

## 4 Conclusion

Harmonic analysis studies of the changes in the pulse wave in response to the acupuncture treatment using the self-comparison method. These findings confirm that harmonic analysis of the pressure pulse is a useful method to quantitatively study and quantify the efficacy of acupuncture treatment on TBI and stroke patient.

## Author contributions

Conceptualization: TJH, HPC, TCW. Methodology: SHW, JKC. Software: SHW, GCW. Validation: TCW, TJH. Formal analysis: SHW, GCW, TCW. Investigation: JKC, WTT, SZL,TJH. Resources: JKC, SZL, TJH. Data curation: JKC, SHW. Writing – Original Draft: JKC, SHW. Writing – Review & Editing: TCW, TJH, HPC. Visualization: SHW. Supervision: TJH. Project administration: 10.13039/501100015640TJH, JKC, SHW. Funding acquisition: TJH, JKC.

## Funding

This research was supported by 10.13039/501100022298Hualien Tzu Chi Hospital (TCRD109-35)

## Ethical statement

This research was reviewed and approved by the institutional review board of Hualien Tzu Chi Hospital (registration number IRB108-200-B). Informed consent was obtained from all participants.

## Data availability

The data are not publicly available due to confidentiality under Taiwan law.

## Declaration of competing interest

The authors declare that they have no conflicts of interest.
