# Supplementary material for: Using radial pulse wave as hemodynamic measurements to quantify effects of acupuncture therapy for patients with traumatic brain injury and ischemia stroke
Source: J Tradit Complement Med. 2022 Sep 1;12(6):594–8. doi: 10.1016/j.jtcme.2022.08.005 (PMC9618386; doi:10.1016/j.jtcme.2022.08.005)
Supplement: Multimedia component 1 [file mmc1.docx]

**Supplementary : additional material**

1. Glasgow Coma Scale (GCS) Assessment

The GCS was based on three different aspects of response - eye open (1:None, 2:To pressure, 3:To speech, 4:Spontaneous), verbal response (1:None, 2:Sounds, 3:Words, 4:Confused, 5:Orientated) and motor response (1:None, 2:Extension, 3:Abnormal flexion, 4:Normal flexion (withdrawal), 5:Localising, 6:Obeying commands).^4^

2. The variation coefficient of nth harmonic amplitude (CnCV) definition

The variation coefficient of nth harmonic amplitude (CnCV) are defined by the following equation (as shown in figure S1):

$\mathrm{Cn}_{\mathrm{avg}}=\frac{1}{M}\sum_{m=1}^{M} \mathrm{Cn}_{m}=\frac{1}{M}\sum_{m=1}^{M} \frac{A_{n,m}}{A_{0,m}}$ (2)

$A_{n,m}e^{-j\theta_{n,m}}=\frac{2}{L}\sum_{k=1}^{L} x_{m} (k)e^{-j2\pi\frac{k*2}{N}}$ (3)

$\sigma_{\mathrm{cn}}=\sqrt{\frac{\sum_{m=1}^{M} (\frac{A_{n,m}}{A_{0,m}}{-\mathrm{Cn}_{\mathrm{avg}})}^{2}}{M-1}}$ (4)

$CnCV= \frac{\sigma_{\mathrm{cn}}}{\mathrm{Cn}_{\mathrm{avg}}}$ (5)

where

- $A_{n,m}$ and $\theta_{n,m}$ are the absolute amplitude and phase of the nth harmonic of the mth radial pulse in the 12 second radial pulse measurement.
- $A_{0,m}$ is the average of the mth radial pulse.
- x_m_ (k) is the kth discrete sampled data point in the mth pulse signal.
- L is the total number of data points in x_m_ (k).
- M is the total number of pulses in one measurement.

**Table S1**

|  | | At the beginning of joined | Before discharge |
| --- | --- | --- | --- |
| Demographic characteristics | |  |  |
| N | 12 | | |
| Age (years) | 63.5 ± 16.2 | | |
| Male (%) | 8 (66.7%) | | |
| BMI (kg/m2) | | 23.0 ± 3.1 | 23.0 ± 3.2 |
| Blood Pressure index | |  |  |
| SBP (mm-Hg) | | 138 ± 16 | 144 ± 21 * |
| DBP (mm-Hg) | | 73 ± 14 | 77 ± 11 |
| HR (bpm) | | 83 ± 12 | 81 ± 12 |
| Glasgow Coma Scale | | 11.4 ± 3.1 | 12.1 ± 3.2 |
| Harmonic index | |  |  |
| C1 | | 0.904 ± 0.032 | 0.955 ± 0.028 |
| C2 | | 0.516 ± 0.043 | 0.556 ± 0.030 |
| C3 | | 0.310 ± 0.037 | 0.336 ± 0.030 |
| C4 | | 0.182 ± 0.015 | 0.189 ± 0.009 |
| C5 | | 0.170 ± 0.010 | 0.162 ± 0.010 |
| C6 | | 0.101 ± 0.012 | 0.105 ± 0.013 |
| C7 | | 0.063 ± 0.006 | 0.078 ± 0.009 * |
| C8 | | 0.047 ± 0.005 | 0.057 ± 0.007 * |
| C9 | | 0.032 ± 0.005 | 0.037 ± 0.005 * |
| C10 | | 0.023 ± 0.003 | 0.030 ± 0.005 * |
| P10 | | 3.049 ± 0.656 | 1.485 ± 0.464 * |
| BMI= Body Mass Index, SBP= Systolic blood pressure, DBP= Diastolic blood pressure, HR= heart rate. Cn= nth Harmonic proportions, P10=10th harmonic phase, * P < 0.05 compared with control | | | |

Table s1 The clinical characteristics and harmonic index of patients with a 3-week course acupuncture treatment


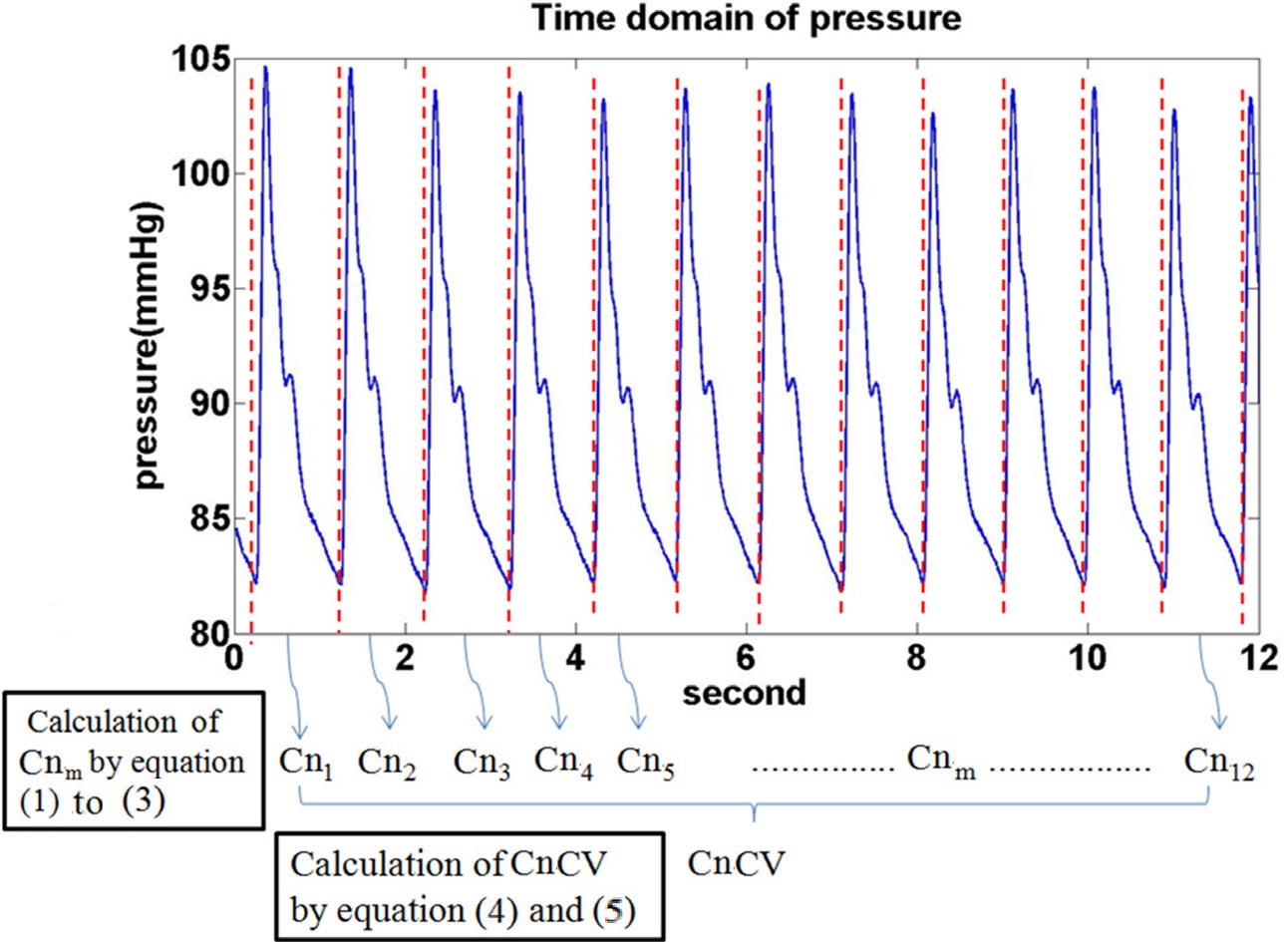


Fig. S1. The calculation of Cn and CnCV using a 12-second radial pulse wave at a sampling rate of 400 Hz.
